# A Multicenter Investigation on the Incidence and Risk Factors of Wound Dehiscence Following Surgical Treatment of Metastatic Spinal Tumors: The Korean Society of Spinal Tumors Multicenter Study (KSST 2023-01)

**DOI:** 10.3390/jcm14051464

**Published:** 2025-02-21

**Authors:** Jin-Sung Park, Dong-Ho Kang, Jae Hwan Cho, Young-Hoon Kim, Han-Dong Lee, Sam Yeol Chang, Sang-Min Park, Se-Jun Park

**Affiliations:** 1 Department of Orthopedic Surgery, Spine Center, Samsung Medical Center, Sungkyunkwan University School of Medicine, Seoul 06351, Republic of Korea; paridot@hanmail.net (J.-S.P.);; 2 Department of Orthopedic Surgery, Asan Medical Center, University of Ulsan College of Medicine, Seoul 05505, Republic of Korea; 3 Department of Orthopedic Surgery, Seoul St. Mary’s Hospital, Catholic University, Seoul 06591, Republic of Korea; 4 Department of Orthopedic Surgery, Ajou University School of Medicine, Suwon 16499, Republic of Korea; 5 Department of Orthopedic Surgery, Seoul National University Hospital, Seoul 03080, Republic of Korea; hewl3102@gmail.com; 6 Spine Center and Department of Orthopedic Surgery, Seoul National University College of Medicine, Seoul National University Bundang Hospital, Sungnam 13605, Republic of Korea

**Keywords:** metastatic spinal tumor, wound dehiscence, risk factor, survival analysis, multicenter study

## Abstract

**Background:** Wound dehiscence is a common complication in metastatic spinal tumor surgery, but its risk factors remain unclear. **Methods:** This retrospective, multicenter study included patients who underwent surgical treatment for metastatic spinal tumors between 2020 and 2022. Data on patient demographics, primary tumor type, comorbidities, laboratory values, surgical details, and the use of radiation therapy, chemotherapy, and steroids were collected. Univariate and multivariate analyses were performed to identify the risk factors associated with wound dehiscence, and survival analysis was conducted based on wound dehiscence. **Results:** Among the 277 patients included, 32 (11.6%) experienced wound dehiscence, with an average time to onset of 37.1 ± 24.3 days. Of these patients, 11 patients with wound infections required revision surgery under general anesthesia, whereas 21 patients underwent localized revision surgery. Univariate analysis identified diabetes (*p* = 0.002), hyperlipidemia (*p* = 0.026), surgical length (*p* = 0.008), and preoperative chemotherapy within 30 days before surgery (*p* = 0.007) as significant risk factors. On multivariate analysis, independent predictors included diabetes (OR: 4.02, 95% CI: 1.66–9.72, *p* = 0.002), surgical length (OR: 1.25, 95% CI: 1.02–1.52, *p* = 0.029), and preoperative chemotherapy within 30 days (OR: 3.75, 95% CI: 1.55–9.10, *p* = 0.003). Preoperative and postoperative radiation therapy did not significantly influence wound dehiscence. Additionally, there was no significant association between wound dehiscence and 90-day mortality or overall survival. **Conclusions:** This study highlights diabetes, surgical length, and preoperative chemotherapy within 30 days as significant predictors of wound dehiscence following metastatic spinal tumor surgery.

## 1. Introduction

The spine is the most common osseous metastasis site, with approximately one-third of patients with cancer experiencing spinal metastases [1,2,3]. Advances in innovative treatments, including targeted drug therapies, immunotherapy, and gene therapy, along with the widespread adoption of multidisciplinary care, have led to an increase in the average life expectancy of patients diagnosed with cancer [4,5]. Consequently, more aggressive treatments are being considered for spinal metastases, with a focus on longevity. In certain cases, even with limited life expectancy, surgical treatment is considered depending on the patient’s general condition and primary cancer type [6].

Surgical treatment is considered in patients with pathological fractures with instability and cord compression caused by spinal metastasis [7,8]. Despite the benefits of surgery in improving patients’ quality of life, complications such as wound dehiscence, infection, neurological deficits, vascular injuries, instrumentation failure, and dural tears occur in 19–28% of cases [9,10]. Among these complications, wound dehiscence, including infection, is the most common complication, with an incidence as high as 20% [11,12]. It is crucial to address this issue, as wound dehiscence can delay the systemic treatment of the primary cancer after surgery [13].

Previous studies have identified medical comorbidities, general condition, adjuvant therapies, such as chemotherapy and radiotherapy, extent of surgery, and blood loss as risk factors for wound dehiscence [14,15,16,17]. However, some studies have reported conflicting results regarding these risk factors, likely owing to various patient-specific factors. Radiotherapy before and after surgery affects wound healing by reducing vascularity, increasing fibrosis, and causing cell damage in the soft tissues, thus leading to an increased risk of wound dehiscence and infection [16,18]. However, some studies have reported that it does not increase the risk of wound complications [19]. The debate over these risk factors continues in the literature, with many studies having small sample sizes, conducted in single institutions, and including long follow-up periods of more than 10 years, which may introduce various biases.

Therefore, the purpose of this study was to analyze the incidence and risk factors of wound dehiscence after metastatic spinal tumor surgery, utilizing recently refined data from a multicenter cohort, and to assess the effect of wound dehiscence on patient survival.

## 2. Materials and Methods

This study was a retrospective, multicenter, observational cohort study conducted in five tertiary hospitals, all of which are members of the Korean Society of Spinal Tumors (KSST) and share treatment methods for spinal metastasis. This study was approved by the local institutional review board and conducted in accordance with the ethical standards of the Declaration of Helsinki. The requirement for informed consent was waived due to the retrospective nature of the data.

Considering advancements in multidisciplinary cancer treatment technologies, this study focused on patients who underwent surgical treatment for spinal metastases between 2020 and 2022. The indications for surgical treatment were carefully determined during a multilateral, inter-department conference based on the following criteria: (1) refractory pain despite conservative treatment and (2) neurological deterioration or the potential for neurological deficits with spinal column instability. Additionally, patients who received sequential adjuvant therapy in medical oncology or radiation oncology after surgical treatment and were available for follow-up for at least three months were included in the study. Surgeries involving minimal wound incisions, such as percutaneous fixation, were excluded. The predefined data forms required for the study and the definition of wound dehiscence were shared among the participants. Patients with insufficient information or those who did not meet the criteria were excluded.

Patient demographics and underlying diseases were identified through medical records, and preoperative general condition was assessed using the Karnofsky Performance Scale (KPS), American Society of Anesthesiologists (ASA) grade, and ambulation status. The origin of the primary malignancy was determined, and the modified Tokuhashi score was used to evaluate the tumor burden at the time of surgery.

Surgical methods were categorized into four types based on operative records: open fixation only, laminectomy and fixation, partial corpectomy and fixation, and total corpectomy and fixation. The surgical length was defined as the segmental level exposed for fixation. Anesthetic records were reviewed to obtain data on the surgical time, blood loss, and transfusion status. The preoperative laboratory results were obtained. To assess the influence of palliative therapy, the timing of preoperative chemotherapy and radiotherapy was documented and analyzed at 15, 30, and 90 days.

During the follow-up period, wound dehiscence was defined as cases where continuous discharge required resuturing before stitch removal or reopening of the wound after stitch removal that necessitated resuturing. For wounds requiring resuturing, wound cultures were performed, and the presence of the identified pathogens was classified as infection-associated wound dehiscence. The study analyzed potential risk factors by comparing patients who experienced wound dehiscence with those who did not and evaluated differences in 90-day mortality and overall survival between the two groups.

Data are presented as frequencies and percentages for categorical variables and as means and standard deviations for continuous variables. Continuous data were analyzed using *t*-tests, and categorical data were analyzed using chi-square tests or Fisher’s exact test. Furthermore, 90-day mortality between the two groups was analyzed using chi-square tests. The overall survival differences between the two groups were assessed with the log-rank test using Kaplan-Meier survival analysis. Stepwise logistic regression analyses were performed using all variables that were significant (*p* < 0.10 in the univariate analyses) to identify the risk factors. Statistical analyses were performed using SPSS (version 27.0.0; IBM Corp., Armonk, NY, USA). Statistical significance was set at *p* < 0.05.

## 3. Results

### 3.1. Demographic Data

This study included 277 patients, with 175 males and a mean age of 60.0 ± 10.6 years. Lung cancer was the most common primary malignancy (81 patients, 29.2%), followed by liver (59 patients, 21.3%) and kidney (24 patients, 8.7%) cancers. The preoperative KPS scores categorized 58 patients as having poor performance (10–40), 125 with moderate performance (50–70), and 94 with good performance scores (80–100). The mean modified Tokuhashi score was 6.2 ± 2.7. Posterior decompression and fixation were the most commonly used procedures. Preoperative therapies included chemotherapy in 128 patients and radiotherapy in 127 patients, whereas postoperative chemotherapy and radiotherapy were administered to 147 and 152 patients, respectively. The mean overall survival was 19.9 ± 1.3 months, and 35 patients died within 90 days. Detailed results are presented in Table 1.

### 3.2. Incidence of Wound Dehiscence

**Figure 1:**
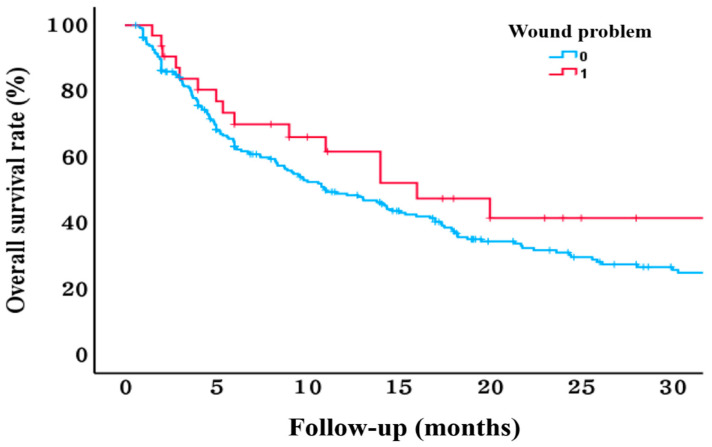
The Kaplan–Meier survival curve shows no significant differences in the overall survival between patients with wound dehiscence and those without wound dehiscence.

Postoperatively, wound dehiscence occurred in 32 patients (11.6%), with a mean onset of 37.1 ± 24.3 days after surgery. Of these cases, 11 involved infections requiring resuturing under general anesthesia, whereas the remaining 21 underwent resuturing under local anesthesia. Regarding survival outcomes, 6 of the 32 patients (18.8%) with wound dehiscence died within 90 days compared to 43 of the 245 patients (17.6%) without wound dehiscence, showing no significant statistical difference. The overall survival was 19.9 ± 2.8 months in patients with wound dehiscence and 19.4 ± 1.4 months in those without wound dehiscence, without statistical significance (Table 2) (Figure 1).

### 3.3. Risk Factors Affecting Wound Dehiscence

Univariate analysis revealed no significant differences between the groups in terms of age, sex, BMI, or smoking status. Diabetes mellitus was significantly more prevalent in the wound dehiscence group (34.4%) than in the non-wound dehiscence group (13.1%) (*p* = 0.002), as was hyperlipidemia (15.6% vs. 5.3%, *p* = 0.026). Other comorbidities showed no significant differences between the groups. General condition indicators such as KPS, ASA grade, and pre- or postoperative ambulation also showed no significant differences. Similarly, the distribution of primary malignancies based on the Tomita scoring system and the modified Tokuhashi score did not differ significantly (Table 3).

Preoperative laboratory findings, surgical methods, operative time, intraoperative blood loss, and transfusion requirements were not significantly different between the groups. However, surgical length was significantly longer in the wound dehiscence group (4.4 ± 1.2) compared to the non-wound dehiscence group (3.7 ± 2.0, *p* = 0.008) (Table 4).

Regarding palliative therapy, surgery within 30 days of completing preoperative chemotherapy was significantly more common in the wound dehiscence group (31.3%) than in the non-wound dehiscence group (13.1%; *p* = 0.007). No significant differences were observed between the 15-day or 90-day intervals. Preoperative radiotherapy, postoperative chemotherapy, and radiotherapy showed no significant differences across the 15-day, 30-day, or 90-day intervals. The use of preoperative steroids to prevent neurological deterioration did not differ significantly between the groups (Table 5).

In the multivariate analysis, the presence of diabetes mellitus (OR 4.02; 95% CI 1.66–9.72, *p* = 0.002), surgical length (OR 1.25; 95% CI 1.02–1.52, *p* = 0.029), and preoperative chemotherapy within 30 days (OR 3.75; 95% CI 1.55–9.10, *p* = 0.003) were identified as independent risk factors for postoperative wound dehiscence (Table 6).

## 4. Discussion

Previous studies have reported risk factors associated with wound-related complications following surgery for spinal metastases [10,11,14,17,19,20,21]. Most of these studies were conducted at a single institution or included patients who were treated more than a decade ago [10,14,19,21]. However, advancements in cancer treatment through multidisciplinary approaches have progressed rapidly in recent years, potentially resulting in differences in indications and treatment methods for patients treated a decade ago [4]. Therefore, this study focused on recent patient incidents from tertiary hospitals affiliated with the KSST, which share treatment strategies for spinal metastases.

Findings from this study demonstrate that 32 patients (11.6%) experienced wound dehiscence after spinal metastasis surgery, with a mean occurrence of 37.1 days postoperatively. Among them, 11 patients had concomitant infections and underwent wound resuturing under general anesthesia, whereas the remaining 21 patients underwent resuturing under local anesthesia. The incidence of infection in the wound dehiscence group (34.4%, 11 of 32) was significantly higher than that in the non-dehiscence group (0.8% 2 of 245). Although it remains unclear whether wound dehiscence or infection occurs first, this study highlights their close associations. Patients with concomitant infections often experience recurrent wound dehiscence after resuturing under local anesthesia, ultimately requiring revision surgery under general anesthesia. Considering that patients with metastatic cancer often present with elevated baseline C-reactive protein levels, advanced age, and chemotherapy-induced immunosuppression, signs of infection may not always be apparent [22]. Therefore, wound culture is recommended for patients with spinal metastases and wound dehiscence, even in the absence of clear signs of infection.

In this study, no significant differences in 90-day mortality or overall survival were observed based on the occurrence of wound dehiscence in patients with spinal metastases. Despite one-third of wound dehiscence cases requiring revision surgery under general anesthesia, such complications did not affect overall survival. Recent studies on survival after surgery for spinal metastases have highlighted the importance of general condition indicators, such as KPS or ambulatory status, over traditional prognostic factors, such as the presence of visceral or other bone metastases included in the Tokuhashi scoring system [23,24,25]. In this study, factors reflecting general condition, such as KPS, ASA grade, and ambulation status, were not identified as risk factors for wound dehiscence. Similarly, the primary malignancy site and modified Tokuhashi scores assessing the tumor burden showed no significant association with wound dehiscence. These findings suggest that subjective assessments of general condition and tumor burden may have limited relevance in the wound healing process.

In this study, factors identified as independent risk factors for wound dehiscence, such as diabetes mellitus, surgical length, and preoperative chemotherapy performed within 30 days, were closely associated with the pathophysiological mechanisms of wound healing. Numerous studies have reported diabetes mellitus as a risk factor for postoperative wound dehiscence not only in the musculoskeletal system of orthopedics but also in various surgical fields [26,27]. Diabetes impairs wound healing by creating a favorable environment for bacterial growth due to vascular and immune system dysfunction, as well as by compromising fibroblast function and collagen synthesis [28,29]. A previous study using codes-in-claims data reported that decompression surgery increases wound complication risks by 1.34 times, and surgeries involving instrumentation increase risks by 2.5 times compared with vertebroplasty in patients with spinal metastases [20]. However, such claims-based studies cannot provide detailed information about the surgical extent. In contrast, this study employed stringent chart reviews to analyze a wide range of surgical factors, revealing no significant differences in wound dehiscence based on surgical methods such as total corpectomy, partial corpectomy, or decompression alone. Additionally, the operative time, intraoperative bleeding, and transfusion did not influence wound dehiscence. The only significant factor was surgical incision length, which has been previously reported as a risk factor for wound reoperation after spinal metastasis surgery [10]. These results suggest that careful consideration of incision length is critical when determining the surgical approach for spinal metastases.

Radiotherapy remains one of the most debated factors in the analysis of the risk of wound complications after metastatic tumor surgery. Earlier studies have reported that preoperative and postoperative radiotherapy increased wound-related complications [18,21]. Radiation impairs fibroblast growth and function, suppresses neovascularization, and affects wound healing [30,31]. However, recent studies suggested that radiation therapy administered before or after surgery does not result in significant wound complications [19]. Advances in radiation techniques, such as hyperfractionation and conformal radiotherapy or stereotactic body radiation therapy, have been shown to reduce normal tissue injury [32]. This study evaluated the risk of wound dehiscence associated with radiotherapy based on the timing of radiotherapy, categorized as 15, 30, or 90 days before or after surgery. Radiotherapy was not identified as a significant risk factor at any time point. The study cohort comprised patients who underwent surgery after 2020, suggesting that many of them likely benefited from advanced radiation techniques designed to minimize the impact on normal tissues. In contrast, chemotherapy administered within 30 days of surgery was significantly associated with an increased risk of wound dehiscence. A previous study also reported that chemotherapy administered within three weeks before surgery increases the risk of wound complications [10]. Chemotherapy affects the cellular and molecular mechanisms of the dermal cells and macrophages involved in wound healing [33]. Therefore, spinal metastasis surgery should be scheduled for at least 30 days after the completion of chemotherapy.

Additional preventive measures could be considered for high-risk patients. Strategies such as intensive wound monitoring and the use of local antimicrobial agents may help reduce the risk. For example, topical vancomycin powder application to the surgical field is a potential adjunct to reduce postoperative infection rates; however, its effectiveness remains inconclusive [34].

This study has some limitations. First, despite being a multicenter study conducted under a preplanned framework by major centers of the KSST, there is inherent variability in treatment methods among different institutions. However, the inclusion of diverse patient populations from multiple centers strengthens the generalizability of our findings. Second, although this was a multicenter study, the sample size was relatively small. This may have limited the statistical power of the subgroup analyses. Nevertheless, the study included only patients recently treated over two years since 2020, with comprehensive data obtained through extensive chart reviews capturing all possible risk factors for wound complications. Despite being a multicenter study, the number of patients with wound dehiscence (n = 32) may be insufficient to fully assess some variables, limiting the statistical power of the analysis. A larger sample size in future studies could provide more robust and precise conclusions regarding risk factors for wound dehiscence following metastatic spinal tumor surgery. Third, this study did not include detailed information on radiotherapy parameters, such as dosage, duration, and fractionation methods, due to variability in data collection across institutions. As these factors may influence wound healing, future studies should aim to incorporate detailed radiotherapy protocols to provide a more precise analysis of its impact on postoperative wound complications. Lastly, this study did not systematically collect data on infection prevention strategies, including the use of prophylactic antibiotics. While most participating hospitals administer a standard prophylactic antibiotic regimen—initiating cefazolin 30 min before surgery and continuing for 24 h postoperatively—variations in practice may exist. Future studies should incorporate detailed antibiotic therapy data to better assess its role in preventing wound infections.

## 5. Conclusions

In this study, wound dehiscence occurred in 32 of the 277 patients (11.6%) after spinal metastasis surgery, with a mean onset of 37.1 days postoperatively. Among the 32 patients, 11 (34.4%) experienced wound infections requiring revision surgery under general anesthesia. However, the occurrence of wound dehiscence did not affect 90-day or overall survival. The independent risk factors for wound dehiscence were diabetes mellitus, surgical length, and chemotherapy within 30 days prior to surgery.

## Figures and Tables

**Table 1:** Baseline demographics.

Variables	Number of Patients (n = 277)
Age at spinal surgery (years)	60.0 ± 10.6
Sex	
Male	175 (63.2)
Female	102 (36.8)
Primary malignancy site factor ^†^	
Lung	81 (29.2)
Liver	59 (21.3)
Kidney	24 (8.7)
Breast	21 (7.6)
Colorectal	14 (5.1)
Prostate	11 (4.0)
Other	67 (24.1)
Preoperative KPS	
10–40	58 (21.0)
50–70	125 (45.1)
80–100	94 (33.9)
Modified Tokuhashi score	6.2 ± 2.7
Surgical procedure type ^‡^	
1	34 (12.3)
2	156 (56.3)
3	66 (23.8)
4	21 (7.6)
Adjuvant therapy	
Chemotherapy	
Before surgery	128 (46.2)
After surgery	147 (53.1)
Radiotherapy	
Before surgery	127 (45.8)
After surgery	152 (54.9)
Overall survival (mean, months)	19.9 ± 1.3
90-day mortality	49 (17.7)
Wound dehiscence	32 (11.6)
Infection	11 (4.0)

Data are presented as the number (%) or mean ± standard deviation; KPS, Karnofsky Performance Status; ^†^ Thyroid cancer (n = 9), lymphoma (n = 8), multiple myeloma (n = 8), stomach cancer (n = 8), cervical cancer (n = 6), cholangiocarcinoma (n = 6), pancreatic cancer (n = 4), gallbladder cancer (n = 4), melanoma (n = 3), leiomyosarcoma (n = 2), endometrial cancer (n = 2), nasopharyngeal cancer (n = 2), liposarcoma (n = 2), parotid cancer (n = 2), and thymus cancer (n = 1); ^‡^ Surgical procedure type: 1, fixation only; 2, laminectomy and fixation; 3, partial corpectomy and fixation; and 4, total corpectomy and fixation.

**Table 2:** Comparison of overall survival and 90-day mortality between patients with wound dehiscence and those without wound dehiscence.

Variables	Dehiscence (32)	No Dehiscence (245)	*p*-Value
Mortality < 90 days	6 (18.8)	43 (17.6)	0.867
Overall survival (months) Mean (95% CI)	19.9 ± 2.8 (14.4 to 25.3)	19.4 ± 1.4 (16.7 to 22.1)	0.286

**Table 3:** Univariate analysis of potential risk factors associated with patient-related conditions for wound dehiscence following metastatic spinal tumor surgery.

Variables	Dehiscence (N = 32)	No Dehiscence (N = 245)	*p*-Value
Age (yrs)	61.4 ± 11.4	59.8 ± 10.5	0.428
Male sex	19 (59.4)	156 (63.7)	0.635
BMI	23.8 ± 3.4	22.9 ± 3.5	0.155
Smoking	7 (21.9)	54 (22.0)	0.983
Comorbidity			
DM	11 (34.4)	32 (13.1)	0.002
HTN	11 (34.3)	69 (28.2)	0.466
Coronary artery disease	2 (6.3)	10 (4.1)	0.571
Renal disease	0 (0)	6 (2.4)	0.371
Thromboembolic event	1 (3.1)	5 (2.0)	0.692
COPD	1(3.1)	3 (1.2)	0.397
Hyperlipidemia	5 (15.6)	13 (5.3)	0.026
General condition			
KPS	60.6 ± 17.0	58.6 ± 17.6	0.541
ASA grade	2.2 ± 0.4	2.3 ± 0.5	0.331
Preop. Ambulation	18 (56.3)	154 (62.9)	0.469
Postop. Ambulation	21 (65.6)	165 (67.3)	0.845
Primary malignancy site *			0.717
Group A (slow growth)	22 (68.8)	154 (62.9)	
Group B (moderate growth)	3 (9.4)	35 (14.3)	
Group C (rapid growth)	7 (21.9)	56 (22.9)	
Modified Tokuhashi score	6.9 ± 2.7	6.2 ± 2.6	0.117

Data are presented as the number (%) or mean ± standard deviation. * Primary malignancy site: Primary malignancy growth rate using Tomita’s scoring system. N, number; BMI, body mass index; DM, diabetes mellitus; HTN, hypertension; COPD, chronic obstructive pulmonary disease; KPS, Karnofsky Performance Status.

**Table 4:** Univariate analysis of potential risk factors associated with laboratory findings and surgical treatment for wound dehiscence following metastatic spinal tumor surgery.

Variables	Dehiscence (32)	No Dehiscence (245)	*p*-Value
Laboratory factor (immediately before surgery)			
WBC	8.5 ± 5.9	8.0 ± 9.9	0.803
Hb	12.4 ± 2.1	12.3 ± 2.0	0.795
HCT	37.2 ± 6.0	38.3 ± 20.3	0.755
PLT	237.9 ± 106.7	216.4 ± 87.6	0.206
Albumin	3.9 ± 0.7	4.0 ± 0.6	0.229
CRP	2.9 ± 4.4	2.1 ± 3.5	0.517
Cr	0.7 ± 0.2	0.8 ± 0.6	0.448
PT	1.1 ± 0.2	1.0 ± 0.1	0.299
aPTT	35.5 ± 4.7	34.1 ± 4.6	0.351
Surgical factors			
Surgical time (minutes)	225.2 ± 168.8	249.7 ± 155.8	0.408
Surgical length (segment level)	4.4 ± 1.2	3.7 ± 2.0	0.008
Surgical procedure type *			0.691
1	3 (9.4)	31 (12.7)	
2	20 (62.5)	136 (55.5)	
3	8 (25.0)	58 (23.7)	
4	1 (3.1)	20 (8.2)	
Blood loss	687.5 ± 697.9	601.8 ± 654.6	0.490
Transfusion	0.7 ± 1.4	0.9 ± 1.6	0.627

* Surgical procedure type: 1, fixation only; 2, laminectomy and fixation; 3, partial corpectomy and fixation; 4, total corpectomy and fixation. WBC, white blood cell count; Hb, hemoglobin; HCT, hematocrit; PLT, platelet count; CRP, C-reactive protein; Cr, creatinine; PT, prothrombin time; aPTT, activated partial thromboplastin time.

**Table 5:** Univariate analysis of potential risk factors associated with palliative therapy for wound dehiscence following metastatic spinal tumor surgery.

Variables	Dehiscence (32)	No Dehiscence (245)	*p*-Value
Palliative therapy			
Preop. CTx (<15 days)	5 (15.6)	23 (9.4)	0.271
(<30 days)	10 (31.3)	32 (13.1)	0.007
(<90 days)	14 (43.8)	82 (33.5)	0.250
Preop. RTx (<15 days)	1 (3.1)	27 (11)	0.163
(<30 days)	4 (12.5)	39 (15.9)	0.616
(<90 days)	6 (18.8)	64 (26.1)	0.367
Postop. CTx (<15 days)	2 (6.3)	28 (11.4)	0.375
(<30 days)	5 (15.6)	50 (20.4)	0.524
(<90 days)	10 (31.3)	101 (41.2)	0.279
Postop. RTx (<15 days)	3 (9.4)	23 (9.4)	0.998
(<30 days)	9 (28.1)	73 (29.8)	0.846
(<90 days)	17 (53.1)	104 (42.4)	0.252
Steroid Treatment	6 (18.8)	56 (22.9)	0.600

CTx, chemotherapy; RTx, radiotherapy.

**Table 6:** Multivariate analysis of potential risk factors for wound dehiscence following metastatic spinal tumor surgery.

Variables	OR (95% CI)	*p*-Value
DM	4.02 (1.66–9.72)	0.002
Hyperlipidemia	3.11 (0.96–10.12)	0.059
Surgical length (segment levels)	1.25 (1.02–1.52)	0.029
Preop. CTx < 30 days	3.75 (1.55–9.10)	0.003

OR, odds ratio; CI, confidence interval; DM, diabetes mellitus; CTx, chemotherapy.

## Data Availability

Data underlying this article cannot be shared publicly due to the privacy of the individuals who participated in this study. The data may be shared by the corresponding author upon reasonable request.

## Abbreviations

The following abbreviations are used in this manuscript:

KSST	Korean Society of Spinal Tumors
KPS	Karnofsky Performance Scale
ASA	American Society of Anesthesiologists
